# Supplementary material for: HOXB7 Overexpression Leads Triple-Negative Breast Cancer Cells to a Less Aggressive Phenotype
Source: Biomedicines. 2021 May 5;9(5):515. doi: 10.3390/biomedicines9050515 (PMC8148148; doi:10.3390/biomedicines9050515)
Supplement: Supplementary file 1 [file biomedicines-09-00515-s001.zip › biomedicines-1179759-supplementary.pdf]

biomedicines

MDPI

Article

HOXB7 Overexpression Leads Triple-Negative Breast Cancer Cells to a Less Aggressive Phenotype

Supplementary materials

1.Figures

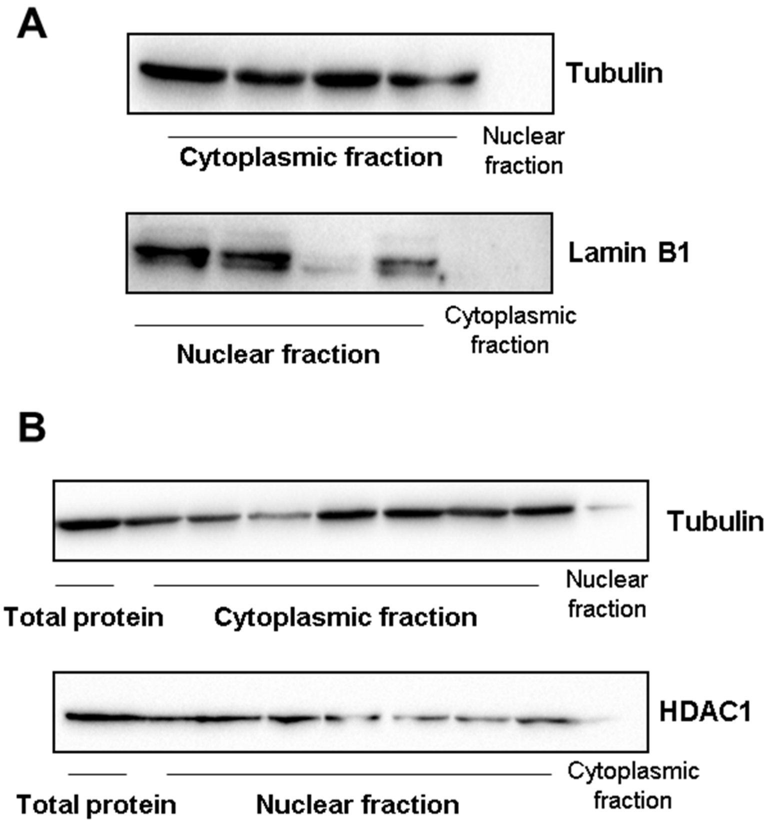

Figure S1. Western Blot representative of the purity of nuclear and cytoplasmic protein fraction extraction. To access the efficiency of the nuclear and cytoplasmic protein extractions, these fractions were also incubated with the loading control of the other condition, as represented. Two different nuclear controls are shown: Lamin B1 (A) and HDAC1 (B). Tubulin is the control protein for the cytoplasmic/total fraction.

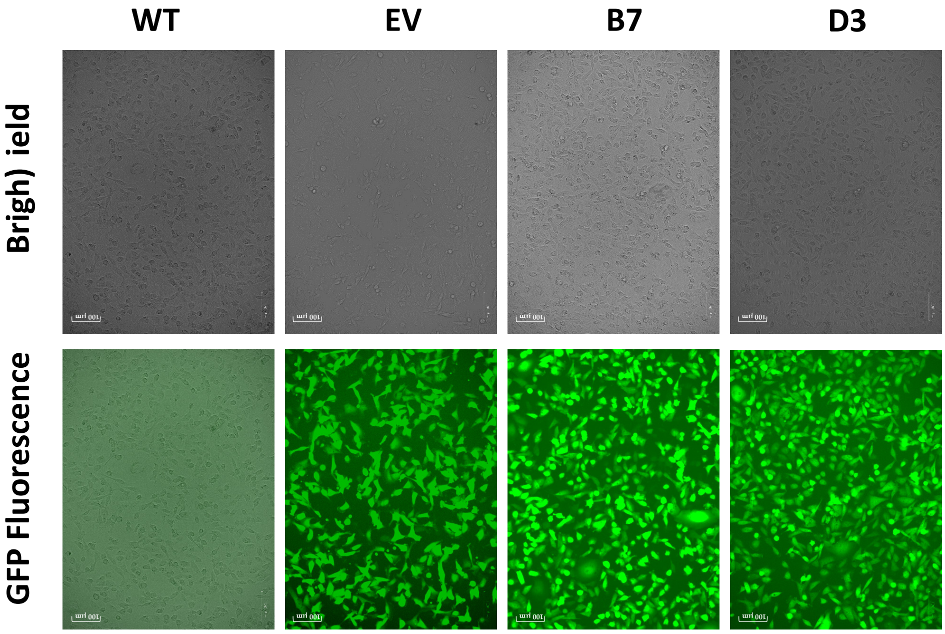

Figure S2. Representative fluorescence image of MDA231 cells after transfection. WT (wild-type cells, not transfected), EV (empty-vector transfected cells), B7 (pool of cells transfected with HOXB7-expression vector) and D3 (clone of cells transfected with HOXB7-expression vector). MDA-MB-231 cells were transfected with plasmid constructs containing the GFP marker in order to access the transfection efficiency and its maintenance. Cells were photographed using the Zoe fluorescent cell imager (Bio-Rad). Scale bar, 100 µm.

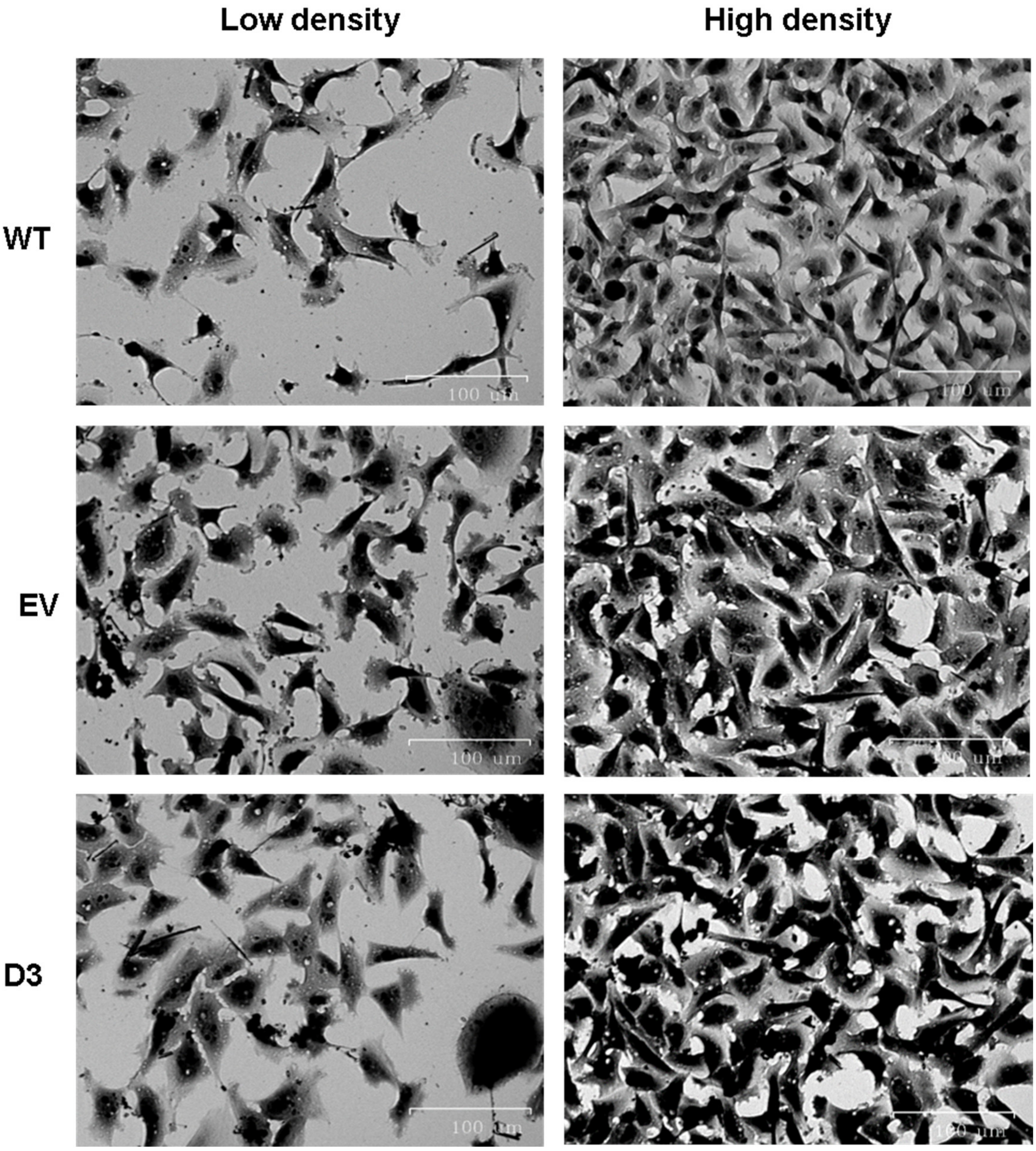

Figure S3. *HOXB7* overexpression effect on MDA231 WT, EV, and D3 cells morphology in 2D cell culture. **WT**, wild-type cells, **EV**, empty-vector transfected cells, and **D3** clone of cells transfected with HOXB7-expression vector. The images are representative of three independent assays.

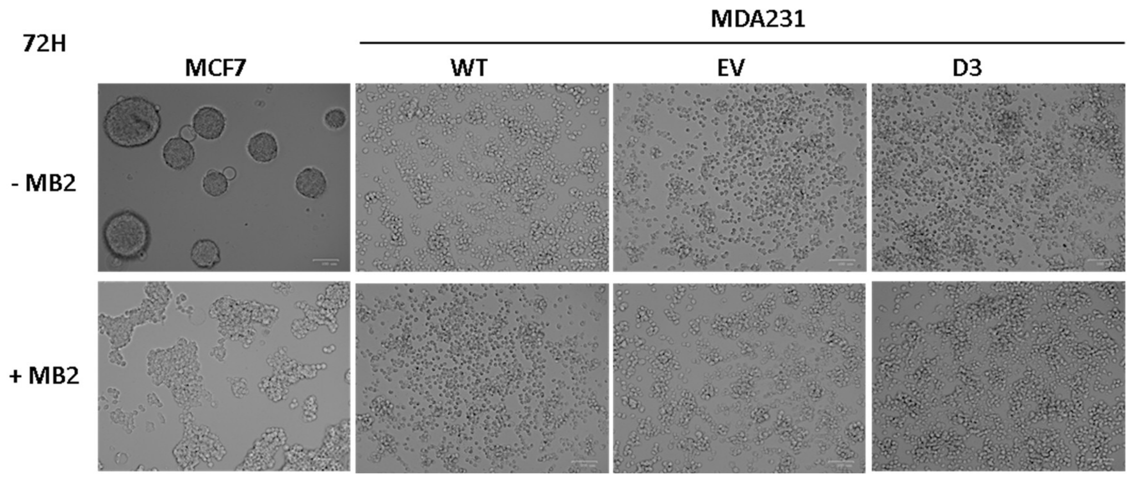

Figure S4. *HOXB7* overexpression effect on MDA231 aggregation capacity. Representative images of the aggregates formed by MCF7 (Positive Control) and MDA231 WT (Negative Control), EV, and D3 cells at 72 h post-plating in the presence (+) and absence (-) of MB2 (anti-CDH1) antibody.

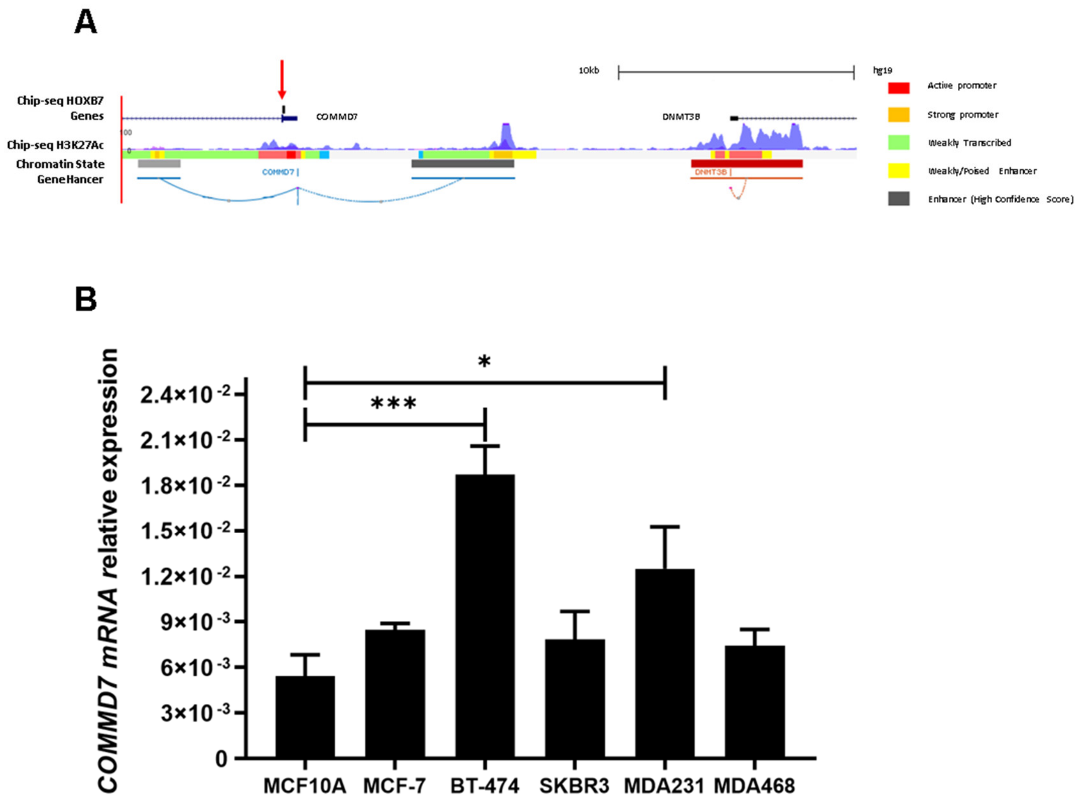

Figure S5. Human genomic landscape and the basal mRNA expression profile of *COMMD7* in breast cancer cell lines. (**A**), human genomic landscape of *COMMD7* and *DNMT3B* that includes an enriched region for *HOXB7* binding site (red arrow). ENCODE data showing the H3K27ac signal distribution that marks active enhancer/promoters and chromatin state segmentation dataset. These results were generated by ENCODE consortium and are available on the Genome Browser at UCSC (http://genome.ucsc.edu/). (**B**), *COMMD7* mRNA expression in MCF7, BT474, SKBR3, MDA231, and MDA468 breast cancer cells in comparison to MCF10A (normal breast cells). Y-axis depicts the ratios of *COMMD7* expression relative to *GAPDH* expression. Statistical analyses by unpaired T-test with Welch's correction. *, p=0.031 and ***, p=0.0009.

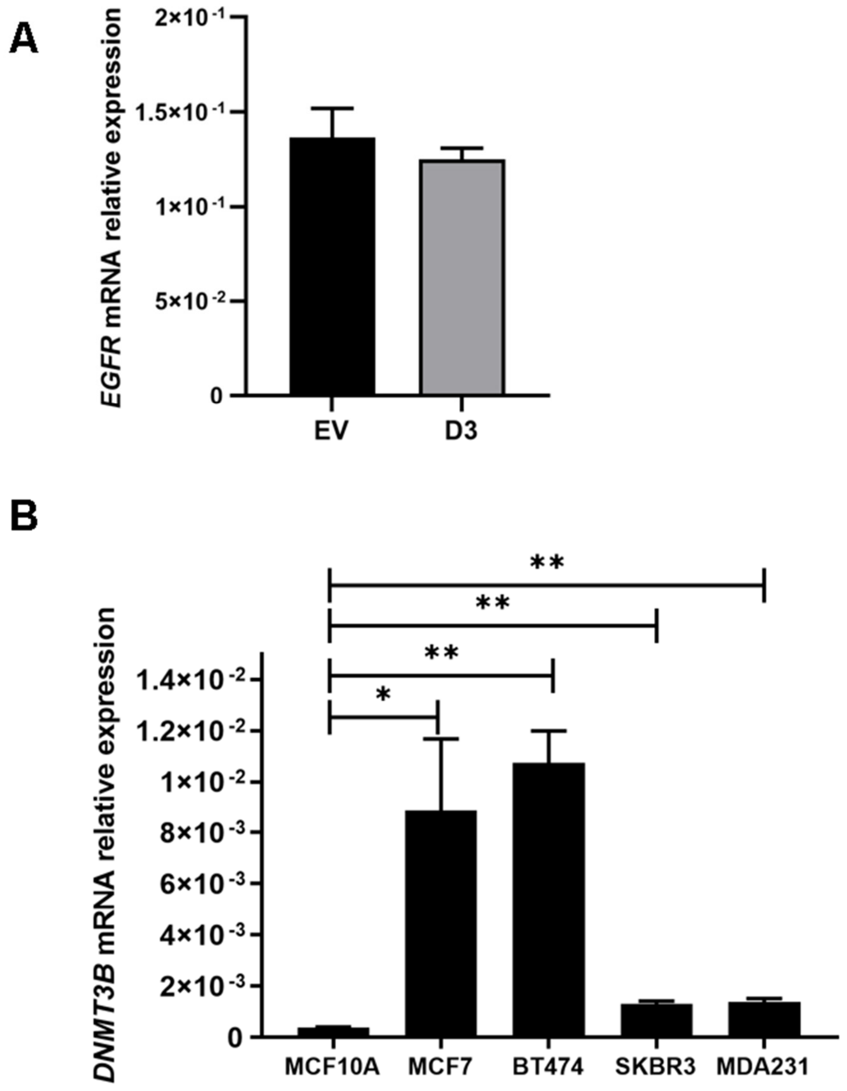

Figure S6. Basal expression profile of *EGFR* in MDA231 EV and D3 cells, and *DNMT3B* in breast cancer cell lines. EV, empty-vector transfected cells and **D3** clone of cells transfected with HOXB7-expression vector**.** (**A**)**,** *EGFR* mRNA expression in MDA231 D3 cells in comparison to EV cells showing no significant expression differences. (**B**), *DNMT3B* mRNA expression in MCF7 (p=0.0347), BT474 (p=0.0048), SKBR3 (p=0.0027) and MDA231 (p=0.0043) breast cancer cells in comparison to MCF10A (normal breast cells). Y-axis depicts the ratios of *EGFR* (**A**) and *DNTM3B (***B**) expression relative to *GAPDH* expression. The graph represents the mean ±SD obtained in three independent experiments. Statistical analyses by unpaired T-test with Welch's correction.

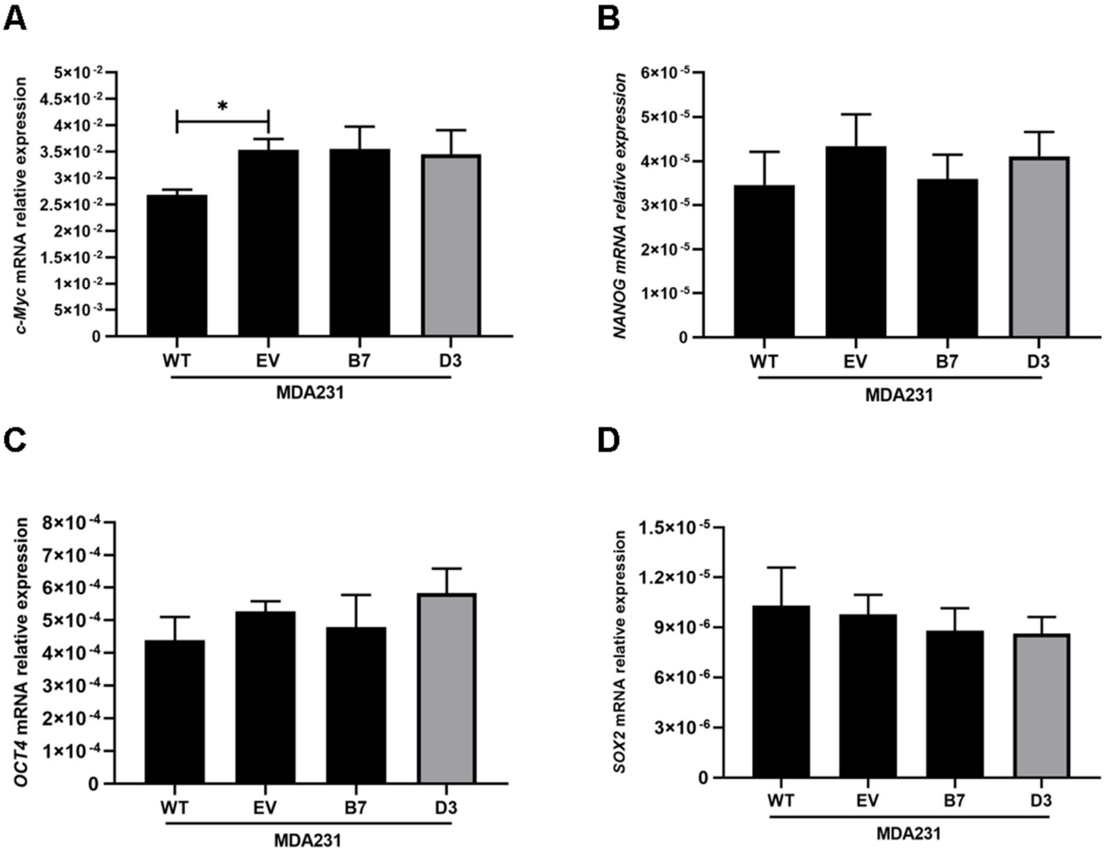

Figure S7. ***c-Myc*** **(A),** ***NANOG*** **(B),** ***OCT4*** **(C), and** ***SOX2*** **(D) mRNA expression in MDA231 cells after transfection. WT,** wild type cells; **EV**, empty-vector transfected cells; **B7,** the pool of cells transfected with HOXB7-expression vector and **D3** clone of cells transfected with HOXB7-expression vector. Y-axis depicts the ratios of the expression of the putative target relative to *GAPDH* expression. The graph represents the mean ±SD obtained in three independent experiments. Statistical analyses by Brown-Forsythe and Welch ANOVA tests (Multiple comparisons) with Games-Howell's correction. *, p=0.026.

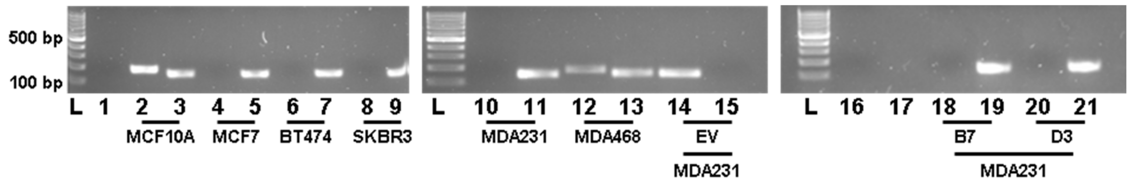

Figure S8. *FGF2* mRNA expression in four breast cancer cell lines (MCF10A, MCF7, BT474, SKBR3, MDA231, and MDA468) including MDA231 cells after transfection. **EV**, empty-vector transfected cells; **B7**, the pool of cells transfected with HOXB7-expression vector and **D3** clone of cells transfected with HOXB7-expression vector. The cDNAs from the cell lines indicated were subjected to a polymerase chain reaction (PCR), using GAPDH and FGF2 primer pairs, and the products were loaded in a 2 % agarose gel. The image is representative of three independent experiments. L, 100 bp DNA ladder; *FGF2* amplification is represented in lanes 2,4,6,8,10,12,15,18 and 20; *GAPDH* amplification is represented in lanes 3,5,7,9,11,13,14,19 and 21; and negative PCR controls is represented in lanes 1 and 16. Note positive *FGF2* expression in MCF10A and MDA 468 cells. Lane 17 is empty.

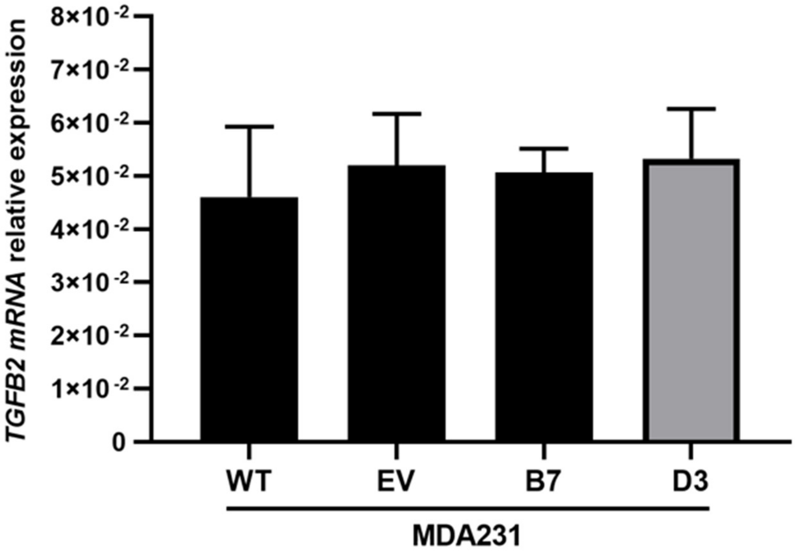

Figure S9. ***HOXB7*** **overexpression effect on** ***TGFB2*** **mRNA expression in MDA231 cells after transfection. WT,** wild type cells; **EV**, empty-vector transfected cells; **B7**, the pool of cells transfected with HOXB7-expression vector and **D3** clone of cells transfected with HOXB7-expression vector**.** Y-axis depicts the ratios of *TGFB2* expression relative to *GAPDH* expression. The graph represents the mean ±SD obtained in at least three independent experiments. No significant expression differences were found by Brown-Forsythe and Welch ANOVA tests (Multiple comparisons) with Games-Howell's correction.

2. Tables

Table S1. qPCR primer sequences, product length, and volume used per reaction used in the present work. The primer sequences not referenced were designed using primer3 online software [6]. *, the annealing temperature for this primer pair is 58°C. Bp, base pair; FW, forward primer; RV, reverse primer, rxn, reaction.

Gene		Sequence (5′-3′)	Product bp	uL /rxn.
COMMD7 **[1]**	FW	GAGCAGCGAATTGGAGAAAGTGG	132	0.5
	RV	TCCATCTCGTGCAGGAAGCTGT		0.5
c-Myc	FW	TACCCTCTCAACGACAGCAG	205	0.5
	RV	CAACATCGATTTCTTCCTCATCTTC		0.5
CTNNB1	FW	GGGTCCTCTGTGAACTTGCT	111	0.5
	RV	TGTAATCTTGTGGCTTGTCCTC		0.5
FGF2* **[2]**	FW	TCAAAGGAGTGTGTGGCTAACCG	176	0.6
	RV	CTGCCCAGTTCGTTTCAGTG		0.6
GAPDH	FW	ACTGGCGTCTTCACCACCAT	142	0.5
	RV	TCTTGAGGCTGTTGTCATACTTC		0.5
HOXB7 **[3]**	FW	TACCCCTGGATGCGAAGCTC	171	0.5
	RV	AATCTTGATCTGTCTTTCCGTGA		0.5
NANOG	FW	TGCAAGAACTCTCCAACATCC	127	0.4
	RV	GCGTCACACCATTGCTATTCT		0.4
OCT4 **[4]**	FW	AACCTGGAGTTTGTGCCAGGGTTT	123	0.4
	RV	TGAACTTCACCTTCCCTCCAACCA		0.4
SOX2 [5]	FW	TTGCTGCCTCTTTAAGACTAGGA	75	0.4
	RV	CTGGGGCTCAAACTTCTCTC		0.4
TGFB2	FW	CCAAAGGGTACAATGCCAAC	128	0.5
	RV	GCAGCAAGGAGAAGCCAGATG		0.5

Table S2. Primers sequences used for ChIP-qPCR reactions, product length, volume used per reaction, and concentration of the necessary additives. The primer sequences not referenced were designed using primer3 online software [6].* For *CDH1* and *CTNNB1* genes the qPCRs were made with two different primer pairs in order to access different regions of the promoter. Bp, base pair; FW, forward primer; RV, reverse primer, rxn, reaction.

		Sequence (5′-3′)	Product bp	uL /rxn.	Additive
CDH1 **CpG***	FW	GTGAACCCTCAGCCAATCAG	108	0.5	Formamide 5%
	RV	CTCACAGGTGCTTTGCAGTTC		0.5	
CDH1 **PROM***	FW	CAGCTTGGGTGAAAGAGTGAG	181	0.35	Formamide 5%
	RV	TCTAGGTGGGTTATGGGACCT		0.35	
COMMD7	FW	CTGGGTGCTTATTGGAAATG	149	0.5	Formamide 5%
	RV	GGACAGGGAAGGGAACAAA		0.5	
CTNNB1 **PROX***	FW	AGTGACAAGTGGAACCAGATAAA	133	0.5	Formamide 5%
	RV	AAGCCCGCAATTCAACAAGT		0.5	
CTNNB1 **DISTAL***	FW	ATAATAAATCAAGCTCTGGGTTT	155	0.5	Formamide 2.5%
	RV	CTCATGTGCCTTCTGGGTTAT		0.5	
DNMT3B **CpG**	FW	GAGCCCGAAGAGGAGAGAAG	149	0.5	Formamide 5%
	RV	CAAGGCAGAGGCCAGGTAAT		0.5	
EGFR **[7]**	FW	CAAGGCCAGCCTCTGAT	174	0.75	Formamide 2.5%
	RV	CCCCTTTCCCTTCTTTTGTT		0.75	
FGF2 **[7]**	FW	GTCATCTGTGGCACCTGCT	199	0.5	Formamide 2.5%
	RV	AGATGAGGATGGGGATAAGGA		0.5	
	RV	AGATGAGGATGGGGATAAGGA		0.5	
TGFB2 **[8]**	FW	GCACACATAATACAGGAGGG	229	0.5	Formamide 5%

RV	CGTTGAGGGAGTGTGGAAAT	0.5

Table S3. Western Blot primary and secondary antibodies usage conditions.

Primary antibody	Blocking solution	Dilution	Secondary antibody	Dilution
HOXB7 (40-2000, Invitrogen)	3% BSA / TBS-T	1 : 500	Rabbit (GE Healthcare, NA934V)	1 : 2000
α-tubulin (Santa Cruz Biotechnology, sc-23948)	5% skim milk / TBS-T	1 : 200000	Mouse (GE Healthcare, NA931V)	1 : 1000
CTNNB1 (Santa Cruz Biotechnology, sc-7963)	3% BSA / TBS-T	1 : 300	Mouse (GE Healthcare, NA931V)	1 : 2000
HDAC1 (Santa Cruz Biotechnology, sc-81598)	3% BSA / TBS-T	1 : 500	Mouse (GE Healthcare, NA931V)	1 : 1000
Lamin B1 (Abcam, ab16048)	5% skim milk / TBS-T	1 : 1000	Rabbit (GE Healthcare, NA934V)	1 : 2000

3. Detailed Information on the Methods

3.1. Cell Culture

The human breast cancer line MDA-MB-231 (MDA231) was authenticated by the Genomics Scientific Platform at i3S using the PowerPlex®16 HS System (Promega Corporation). Detection of the amplified fragments was made with automated capillary electrophoresis using the 3130 Genetic Analyzer (Applied Biosystems) and the assignment of genotypes was performed in GeneMapper software v5.0 (Applied Biosystems). The cells were cultured in Dulbecco's modified Eagle's medium, DMEM 1X (GIBCO, Paisley, UK) supplemented with 10% (V/V) heat-inactivated fetal bovine serum (FBS, Biowest, South American Origin) and 1X antibiotic solution penicillin-streptomycin, pen-strep (Gibco, Grand Island, USA), and maintained at 37°C in a 5% CO_2 humidified atmosphere. Cells were used in experiments upon reaching 70–80% of confluence. The cells generated after the transfection assays were cultured in the same medium as MDA231 cells adding 700μg/mL geneticin (Gibco, Thailand) for the maintenance of cell selection pressure. Note that, for the assays described below, the transfected cells were kept in a complete medium without geneticin.

3.2. Stable Transfection Assay

To generate stable *HOXB7*-overexpressing and control cells, MDA231 were transfected with the pCMV6-AC-GFP-*HOXB7* (Origene, RG204495) or pCMV6-AC-GFP (Origene, PS100010) constructs. Transfections were performed using TurboFectin 8.0 (Origene) reagent according to the manufacturer's instructions. Briefly, MDA231 cells were seeded in a 6-well plate on the previous day in order to get 50-70% confluence on the following day. The complexes formation were made in two different ratios, 1μg DNA: 3μl TurboFectin 8.0 and 1.5μg DNA: 6μl TurboFectin 8.0, and the transfected cells were incubated at 37°C in a 5% CO_2 humidified atmosphere for 24 hours post-plating. After incubation, the cells medium was changed and geneticin (1.2mg/mL) was added for the selection of the stably transfected cells. The GFP-positive/geneticin-resistant cells were then sorted in the FACSAria™ II cell sorter (BD Biosciences). Cells transfected with pCMV6-AC-GFP-HOXB7 plasmid vector were seeded in 96-well plates (1 cell/well) containing 20% FBS in order to achieve the clonal expansion of one cell and the remaining cells were collected in a T25 flask. The cells transfected with the empty vector (pCMV6-AC-GFP) were collected in a T25 flask with the conventional complete medium. Once cells restarted proliferation both in the T25 flask and in the 96-well plate they were kept in the conventional medium containing 700μg/mL geneticin. Three different cell transfectants were obtained and named as Empty Vector (EV), for the cells transfected with the control empty vector (pCMV6-AC-GFP); B7, for the pool of cells transfected with pCMV6-AC-GFP-

HOXB7 vector and overexpressing variable levels of *HOXB7*; D3, for the clone obtained from the 96-well plate sorted cells transfected with pCMV6-AC-GFP-*HOXB7* vector.

3.3. RNA Expression Analyses

The total RNA extraction was performed using TRIzol™ reagent (Ambion, Carlsbad, USA) according to the manufacturer's instructions and adding one more wash with ethanol 75%. After assessment of RNA 260/280nm ratio and concentration using NanoDrop 1000 (Thermo Scientific), 800ng of RNA was subjected to reverse transcription, using the High-Capacity cDNA Reverse Transcription Kit (Applied Biosystems, Vilnius, Lithuania), following the manufacturer's instructions. The qPCRs reactions were performed in duplicates and carried out in the CFX96™ Real-Time PCR Detection System (Bio-Rad). Each 10μL amplification reaction contained 2μL of the respective cDNA diluted 1:4; the indicated quantity of forward and reverse 10μM primers (Supplementary Table 1); 5μL of 2X iTaq™ Universal SYBR Green Supermix (Bio-Rad, USA) and 2μL of DNase/RNase free H_2O. The run conditions were: 95˚C for 3 minutes (min.), 40 cycles of 95˚C for 10 seconds (sec.), and 60˚C for 30sec. followed by the default dissociation curve capture. The qPCR result analyses were performed using the method described by Schmittgen and Livak [9] using the formula: RATIO = E target – (CT sample target gene) /E *GAPDH* – (CT sample GAPDH), in which "E" is the primer pair efficiency previously calculated and *GAPDH* (glyceraldehyde-3-phosphate dehydrogenate) is the reference gene. The primer sequences used for qPCR were synthesized by Sigma Aldrich (Darmstadt, Germany) and are described in Supplementary Table 1.

3.4. ChIP-qPCR Assay

The cells were seeded in cell culture dishes with 15cm in diameter in conventional medium and grown until reaching 80-90% confluence. Then, the DNA / proteins crosslink was performed using 1% formaldehyde solution incubation under agitation for 10min. at room temperature. Next, to stop crosslink, 0.15M glycine was added directly onto the shaking plates and incubated for 5min. at room temperature followed by two washes with cold 1X PBS (Gibco, Paisley, UK). Then, cells were collected in 1.5 mL tubes after cell scrape. The obtained pellets, after 5min. centrifugation at 1000*g* at 4°C, were suspended in Cell lysis buffer (5mM Pipes, 85mM KCl, 0.5% Igepal, 1X Roche cOmplete™ Protease Inhibitor Cocktail, 1μL/mL Trypsin, 10μL/mL PMSF 200mM, 1μL/mL DTT 50mM in MilliQ H_2O) and incubated on ice for 10min. followed by centrifugation of 1000 *g* for 5min. at 4°C. The cell pellet was suspended in Nuclear lysis buffer (50mM Tris, 10mM EDTA, 1% SDS 1X Roche cOmplete™ Protease Inhibitor Cocktail, 1μL/mL Trypsin, 10μL/mL PMSF 200mM, 1μL/mL DTT 50mM in MilliQ H_2O), incubated for 10min. on ice and immediately sonicated (30 cycles, 30 sec. ON/30 sec. OFF, high mode) in the Bioruptor Plus (Diagenode). The sonicated DNA (1μg) fragment sizes were analyzed in a 1% agarose gel with an expected smear under 1000bp with a strong band at 500bp. The DNA quantification was made in Nanodrop 1000 (Thermo Scientific) after the following preparation: 20μl of sonicated samples was mixed in 20μl of 10% Chelex® 100 resin (Bio-Rad, USA), incubated for 5min. at 90°C and centrifuged 15000*g* for 1min. The blank sample was prepared adding 20μl of nuclear lysis buffer to 20μl of 10% Chelex® 100 resin and following the same steps mentioned for the sample preparation. After DNA quantification and gel analyses, the sonicated DNA samples were heated for 5min. at 65°C and centrifuged 15000 *g* for 30 sec. Forty micrograms of DNA *per* Immunoprecipitation (IP) was diluted 1:20 in Dilution buffer (0.01% SDS, 1,1% Triton X-100, 1.2mM EDTA pH 8.0, 16.7mM Tris pH 8.1, 167mM NaCl in MilliQ H_2O) and 4μg of the desired antibody was added. The IPs were incubated overnight at 4°C under rotation. An input control was prepared for each sample that was used diluting, also in a 1:20 ratio, 4μg of each sample (10% of the IP) in Dilution buffer followed by the addition of 3 parts absolute ethanol and overnight precipitation at -80°C. On the day after, considering that 40μl of Dynabeads A/G were used per IP, a mixture

was prepared of 1 volume Dynabeads A and 1 volume Dynabeads G, followed by the beads separation with a magnetic stand, aspiration of the buffer, and the addition of the same initial volume of IP buffer (10% Nuclear lysis buffer, 90% Dilution buffer). Next, the samples with antibodies were centrifuged for 10min. at 12000 *g*, the supernatants were transferred to a new tube and 40µl of Dynabeads preparation was added followed by incubation for 2h at 4°C with rotation. The beads were, then, separated with a magnetic stand and washed (15min. rotation at room temperature followed by beads separation) twice with 1mL Dialysis buffer (2mM EDTA pH 8.0, 50mM Tris pH 8.1, 0.2% Sarkosyl in MilliQ H_2O) and four times with 1 mL wash buffer (0.5 M LiCl, 1% Igepal, 1% Na-deoxycholate, 33.2mM Tris pH 8.1 in MilliQ H_2O). After the last wash, beads were eluted in 150µl Elution buffer (50mM NaHCo3, 1% SDS), heated 15min. at 65°C with vortex each 5min. and the supernatant transferred to a new tube. This step was repeated yielding 300µl of supernatant per sample. The input controls were centrifuged at 12000 *g* for 10min. after the precipitation step and the pellets were washed twice with 70% ETOH (1 mL 70% ETOH and identical centrifugation), eluted with 300µl Elution buffer, and heated 15min. at 65°C with vortex every 5min. To IPs and Input samples 2.4µl Proteinase K (20mg/mL) was added followed by 1h incubation at 55°C at 90*g* and purification with QIAquick PCR purification kit (Qiagen, Hilden, Germany) according to manufacturer's instructions. Samples were suspended in DNase/RNase-free H_2O and stored at -20°C. The ChIP-qPCR reactions were performed using 2µL of the purified sample and the run conditions were: 95˚C for 3min., 40 cycles of 95˚C for 15 sec., 55˚C for 30 sec. and 72˚C for 30 sec. followed by the default dissociation curve capture. The assays analyses were made using the "percent input" method according to Lacazette [10]. The primers sequences used for ChIP-qPCR were synthesized by Sigma Aldrich (Darmstadt, Germany) and are described in Supplementary Table 2.

3.5. Protein Expression Assays

Total protein extraction was obtained collecting the cells pellets after dissociation, suspending them in the lysis solution (0.1% Triton X-100, 0.1% Igepal, 1X cOmplete™ Protease Inhibitor Cocktail [Roche, Germany], PBS 1X [Gibco, Paisley, UK]) and incubating on ice for 20min. with vortex every 5min. After incubation, the tubes were centrifuged at 9000 *g* for 10min. at 4°C and the supernatant transferred to a new tube. An aliquot of the protein extracts was separated for posterior quantification. Samples were stored at -80°C.

Cytoplasmic and nuclear proteins fractions extractions were performed using the ab113474 Nuclear Extraction Kit (Abcam) according to the manufacturer's recommendations. The nuclear proteins fractions were sonicated in Bioruptor Plus (Diagenode) for 3 cycles, 10 seconds ON / 10 sec. OFF at high mode to increase extraction yield. Aliquots of both fractions were separated for posterior quantification. Samples were stored at -80°C.

Protein concentrations were determined using the Pierce Detergent Compatible Bradford Assay Kit (Thermo Scientific, Rockford, USA) following the product data-sheet instructions. The albumin standards were prepared in a working range of 100-1500µg/mL and the samples were quantified in a 1:10 dilution. Both, sample dilutions and albumin standards were prepared in RNase/DNase-free H_2O. Twenty to thirty micrograms of protein lysates were separated on a 12% SDS-PAGE gel prepared with 40% Acrylamide – Bisacrylamide 29:1 (Invitrogen, Carlsbad, USA) and 4X separating buffer (Alfa Aesar, Ward Hill, USA) for the separating gel or 4X stacking buffer (Alfa Aesar, Karlsruhe, Germany) for the stacking gel. After run the proteins were blotted onto a nitrocellulose membrane of the iBlot® gel transfer stacks (Kiryat Shmona, Israel) using the iBlot™ dry transfer system (Life Technologies, Israel). Blots were blocked for 1h at room temperature in 3% BSA/TBS-T (20mM Tris, 137mM NaCl, 0.1% Tween-20) solution or 5% skim milk/TBS-T solution and incubated overnight at 4°C with the primary antibodies, diluted in the respective blocking solutions. Then, membranes were washed four times in TBS-T for 5min., two times in TBS (20mM Tris, 137mM NaCl) for 5min. and incubated with the appropriate horseradish peroxidase-conjugated secondary IgG antibody for 1h30min. at room

temperature. The bolts were washed again, as already described, followed by the detection of the immunoreactive proteins using the Clarity™ Western ECL Substrate (Bio-Rad, USA) in the ChemiDoc Gel Image System (Bio-Rad). The primary and secondary antibodies used in this work are listed in Supplementary Table 3. The assessment of the band's density was made in the ImageLab software (BioRad) using the measures of Tubulin (for total and cytoplasmic protein fractions) and HDAC1/Lamin B1 (for nuclear protein fraction) as loading controls. A representative western blot of the purity of nuclear and cytoplasmic protein fractions extraction is shown in Supplementary Fig. 1.

3.6. MTT Assay with Docetaxel Treatments

Cell viability was determined using MTT assay [3-(4,5-dimethylthiazol-2yl)-2,5-diphenyltetrazolium bromide] (EMD Millipore, China) following the manufacturer's instructions. Cells were seeded in 96-well plates ($1x10^4$ cells/well) and maintained at 37°C in a 5% CO2 humidified atmosphere in complete media supplemented with 5% FBS for 24h. At this time point (24h), the Docetaxel (Sigma-Aldrich, China) treatments with 5nM and 50nM started. The cell viability measures were made by absorbance read at 570nm using the Synergy™ 2 Plate reader (BioTek) at 24h, 48h, 72h, and 96h post-seed. The medium containing Docetaxel or vehicle (ETOH) was changed every 24h to ensure that cells were exposed to the same drug concentration along of time points analyzed. The control cells were treated with the drug vehicle (ETOH) in the volume corresponding to the biggest drug concentration used. An additional assay was made for the measure of the cell viability in a standard medium without ETOH.

3.7. On-Top 3D Cell Culture

For the morphogenesis assay, MDA231 wild-type (WT), EV and D3 cells were dissociated to obtain a suspension containing single cells. The cells ($2×10^3$), suspended in 400μL of completed media containing 3% of Matrigel® Matrix Basement Membrane growth factor reduced (Corning, Bedford, USA), were seeded in 8-well glass chamber slides containing a pre-prepared bed of 30μl Matrigel® Matrix Basement Membrane growth factor reduced, as described by Debnath *et al.* [11] and incubated at 37°C in 5% CO2 humidified atmosphere for 8 days. The cells spheroids morphologies were analyzed and registered every two days in Axiovert 200M inverted fluorescent microscope (Carl Zeiss). On day-4, 100μl of media containing 3% Matrigel® Matrix Basement Membrane growth factor reduced were added to the wells to prevent the effects of medium evaporation and nutrient scarcity.

3.8. Wound Healing Assay

Cells were seeded in 24-well plates at a density of $2×10^5$ cells/well in 500μl of Dulbecco's modified Eagle's medium, DMEM 1X (GIBCO, Paisley, UK) supplemented with 5% (V/V) Charcoal Stripped Fetal Bovine Serum Qualified One Shot™ (Gibco, Mexico), for cells mitosis synchronization, and 1X antibiotic solution penicillin-streptomycin (pen-strep, Gibco™). Forty-eight hours post-seed, to ensure cell synchronization and growth to 90%-100% confluence, a single scratch wound was made in each well using a 200μl disposable pipette tip. The cells were then incubated for 15h in the InCell Analyser 2000 Automated fluorescence widefield HCS microscope for the capture of wound images every 3h. The extent of wound closure was measured using the MRI wound healing tool from ImageJ software [12].

3.9. Invasion Assay

The invasion assay was made using the 24-well plate growth factor reduced Corning® Matrigel® Invasion chambers 8μm pore size (Corning, Bedford, USA) and 24-well Control Inserts 8μm pore size (Corning, Bedford, USA) according to manufacturer's instructions. Briefly, cells ($1 × 10^5$) in 500μl serum-free medium were plated into the upper

chamber and the bottom wells were filled with 750µl complete medium. The cells were incubated at 37°C in a 5% CO_2 humidified atmosphere for 16h. Then, cells in the upper chamber were removed using cotton swabs and the cells invading the bottom of the membrane were fixed with 4% paraformaldehyde for 20min. The nuclei were stained with DAPI (Sigma-Aldrich, Darmstadt, Germany) 1µg/mL plus 1% Triton X-100 in 1X PBS (Gibco, Paisley, UK) for 15min. followed by two washes in Milli-Q H_2O. Ten random fields from each membrane were photographed using the Zoe fluorescent cell imager (Bio-Rad) and the cells' nuclei were counted using the Analyse Particles tool from ImageJ software [12].

3.10. Soft agar Colony Formation Assay

This assay was made as described by Borowicz and colleagues [13]. The cells (1.5 x 10^4/well) were seeded in 6-well plates and incubated for four weeks at 37°C in a 5% CO_2 humidified atmosphere. The colonies, without staining, of nine random fields, were counted using the Zoe fluorescent cell imager (Bio-Rad). MCF7 cells cultured in the same medium as MDA231 cell line were used as a parameter for Luminal behavior analyses.

3.11. Slow Aggregation Assay

This assay was performed according to the protocol described by Boterberg *et al* [14]. Briefly, 2 x 10^4 cells per well were seeded over an agar layer in a 96-well plate with and without the addition of MB2, an anti-CDH1 antibody, as a way to show if the formed aggregates are dependent on the CDH1/CTNNB1 complex functionality. The cells were photographed after 24h, 48h, 72h, and 96h in an Axiovert 200M inverted fluorescent microscope (Carl Zeiss). The MDA231-WT and MCF7 cells were used, respectively, as negative and positive controls of the aggregation capacity. MDA231-WT cells do not express CDH1 and, therefore, they do not aggregate while MCF7 cells, which express high levels of CDH1, form well-defined aggregates when CDH1/CTNNB1 complex is active.

3.12. Statistical Analyses

The statistical differences were determined by unpaired T-test with Welch's correction or by Brown-Forsythe and Welch ANOVA tests using the Prism8 software (GraphPad Software, La Jolla, USA). p -values were considered statistically significant when $p \leq 0.05$. Data were reported as the mean ±SD of three or more independent experiments.

References

1. Shah, F.; Goossens, E.; Atallah, N.M.; Grimard, M.; Kelley, M.R.; Fishel, M.L. APE 1/Ref-1 knockdown in pancreatic ductal adenocarcinoma – characterizing gene expression changes and identifying novel pathways using single-cell RNA sequencing. *Mol. Oncol.* **2017**, *11*, 1711–1732, doi:10.1002/1878-0261.12138.
2. Wu, X.; Chen, H.; Parker, B.; Rubin, E.; Zhu, T.; Lee, J.S.; Argani, P.; Sukumar, S. HOXB7, a Homeodomain Protein, Is Overexpressed in Breast Cancer and Confers Epithelial-Mesenchymal Transition. *Cancer Res.* **2006**, *66*, 9527–9534, doi:10.1158/0008-5472.can-05-4470.
3. Ma, R.; Zhang, D.; Hu, P.-C.; Li, Q.; Lin, C.-Y. HOXB7-S3 inhibits the proliferation and invasion of MCF-7 human breast cancer cells. *Mol. Med. Rep.* **2015**, *12*, 4901–4908, doi:10.3892/mmr.2015.4009.
4. Gehring, W.J. The homeobox in perspective. *Trends Biochem. Sci.* **1992**, *17*, 277–280, doi:10.1016/0968-0004(92)90434-b.
5. Lengerke, C.; Fehm, T.; Kurth, R.; Neubauer, H.; Scheble, V.; Müller, F.; Schneider, F.; Petersen, K.; Wallwiener, D.; Kanz, L.; et al. Expression of the embryonic stem cell marker SOX2 in early-stage breast carcinoma. *BMC Cancer* **2011**, *11*, 1–10, doi:10.1186/1471-2407-11-42.
6. Untergasser, A.; Cutcutache, I.; Koressaar, T.; Ye, J.; Faircloth, B.C.; Remm, M.; Rozen, S.G. Primer3—new capabilities and interfaces. *Nucleic Acids Res.* **2012**, *40*, e115, doi:10.1093/nar/gks596
7. Heinonen, H.; Lepikhova, T.; Sahu, B.; Pehkonen, H.; Pihlajamaa, P.; Louhimo, R.; Gao, P.; Wei, G.; Hautaniemi, S.; Jänne, O.A.; et al. Identification of several potential chromatin binding sites of HOXB7 and its downstream target genes in breast cancer. *Int. J. Cancer* **2015**, *137*, 2374–2383, doi:10.1002/ijc.29616.
8. Liu, S.; Jin, K.; Hui, Y.; Fu, J.; Jie, C.; Feng, S.; Reisman, D.; Wang, Q.; Fan, D.; Sukumar, S.; et al. HOXB7 Promotes Malignant Progression by Activating the TGFβ Signaling Pathway. *Cancer Res.* **2015**, *75*, 709–719, doi:10.1158/0008-5472.can-14-3100.

9. Schmittgen, T.D.; Livak, K.J. Analyzing real-time PCR data by the comparative CT method. *Nat. Protoc.* **2008**, *3*, 1101–1108, doi:10.1038/nprot.2008.73.
10. Lacazette, E. A laboratory practical illustrating the use of the ChIP-qPCR method in a robust model: Estrogen receptor alpha immunoprecipitation using Mcf-7 culture cells. *Biochem. Mol. Biol. Educ.* **2016**, *45*, 152–160, doi:10.1002/bmb.20999.
11. Debnath, J.; Muthuswamy, S.K.; Brugge, J.S. Morphogenesis and oncogenesis of MCF-10A mammary epithelial acini grown in three-dimensional basement membrane cultures. *Methods* **2003**, *30*, 256–268, doi:10.1016/s1046-2023(03)00032-x.
12. Schneider, C.A.; Rasband, W.S.; Eliceiri, K.W. NIH Image to ImageJ: 25 years of image analysis. *Nat. Methods* **2012**, *9*, 671–675, doi:10.1038/nmeth.2089.
13. Borowicz, S.; Van Scoyk, M.; Avasarala, S.; Karuppusamy Rathinam, M.K.; Tauler, J.; Bikkavilli, R.K.; Winn, R.A. The Soft Agar Colony Formation Assay. *J. Vis. Exp.* **2014**, *e51998*, e51998, doi:10.3791/51998.
14. Boterberg, T.; E Bracke, M.; A Bruyneel, E.; Mareel, M.M.; Brooks, S.A.; Schumacher, U. Cell Aggregation Assays. *Metastasis Research Protocols* **2003**, *58*, 033–045, doi:10.1385/1-59259-137-x:033.
